# How to Sustainably Feed a Microbe: Strategies for Biological Production of Carbon-Based Commodities with Renewable Electricity

**DOI:** 10.3389/fmicb.2016.01879

**Published:** 2016-11-28

**Authors:** Caitlyn S. Butler, Derek R. Lovley

**Affiliations:** ^1^Civil and Environmental Engineering, University of MassachusettsAmherst, MA, USA; ^2^Microbiology, University of MassachusettsAmherst, MA, USA

**Keywords:** renewable energy storage, artificial photosynthesis, CO_2_ sequestration, biocommodities, microbial electrosynthesis

## Abstract

As interest and application of renewable energy grows, strategies are needed to align the asynchronous supply and demand. Microbial metabolisms are a potentially sustainable mechanism for transforming renewable electrical energy into biocommodities that are easily stored and transported. Acetogens and methanogens can reduce carbon dioxide to organic products including methane, acetic acid, and ethanol. The library of biocommodities is expanded when engineered metabolisms of acetogens are included. Typically, electrochemical systems are employed to integrate renewable energy sources with biological systems for production of carbon-based commodities. Within these systems, there are three prevailing mechanisms for delivering electrons to microorganisms for the conversion of carbon dioxide to reduce organic compounds: (1) electrons can be delivered to microorganisms via H_2_ produced separately in a electrolyzer, (2) H_2_ produced at a cathode can convey electrons to microorganisms supported on the cathode surface, and (3) a cathode can directly feed electrons to microorganisms. Each of these strategies has advantages and disadvantages that must be considered in designing full-scale processes. This review considers the evolving understanding of each of these approaches and the state of design for advancing these strategies toward viability.

## Introduction

There is a need for strategies to store or effectively utilize the excess energy available when the production of renewable electricity exceeds demand (Lovley and Nevin, 2013; Rosenbaum and Henrich, 2014). A potentially sustainable biological solution would be to feed the electrical energy to a microbe that could either: (1) store the energy in a product that could later be efficiently converted back to electricity when demand was higher; or (2) produce a fuel that could replace fossil fuels; or (3) produce a commodity that otherwise would be made from non-renewable feedstocks such as petroleum.

**Figure 1:**
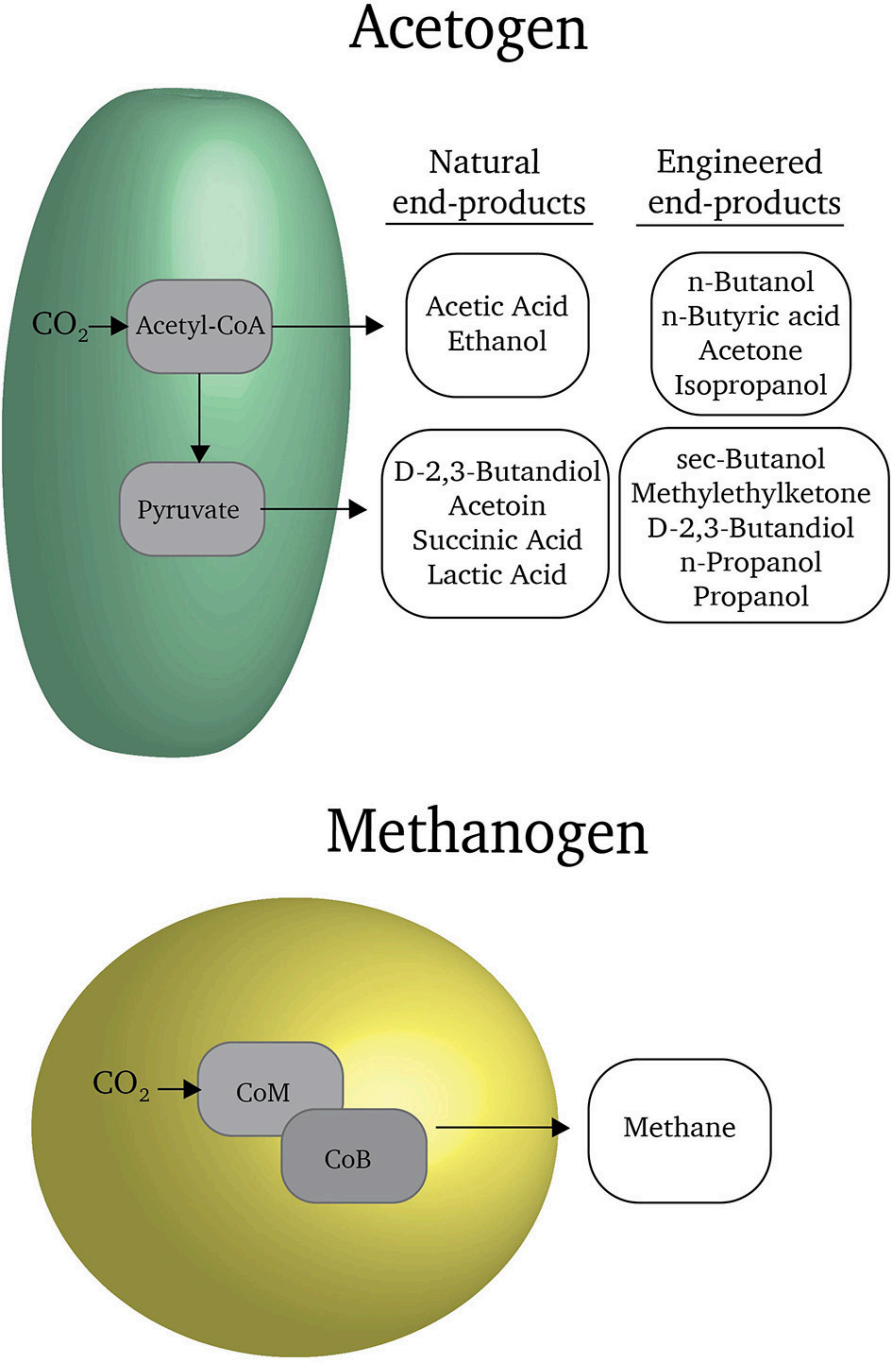
**Reduced carbon products produced by acetogens and methanogens**. Through genetic engineering, the catalog of feasible products can be expanded. Further detail regarding these products is reviewed in Liew et al. (2016).

Microorganisms are masters of organic chemistry and have the potential to directly produce organic fuels and commodities from carbon dioxide (Figure 1). For example, methanogenic microorganisms can reduce carbon dioxide to methane gas (Martin et al., 2013). Acetogenic microorganisms naturally reduce carbon dioxide to more complex molecules with carbon-carbon bonds such as acetate and ethanol (Liew et al., 2016). With some genetic manipulation of metabolic pathways, it is possible to coax acetogens to produce fuels such as butanol and commodities such as butyrate and butanediol (Liew et al., 2016). Typically over 90% of the electrons available in substrates for methanogens and acetogens are recovered in the organic products that they release into the extracellular medium (Lovley and Nevin, 2013). Few of the electrons are diverted into producing more cells.

**Figure 2:**
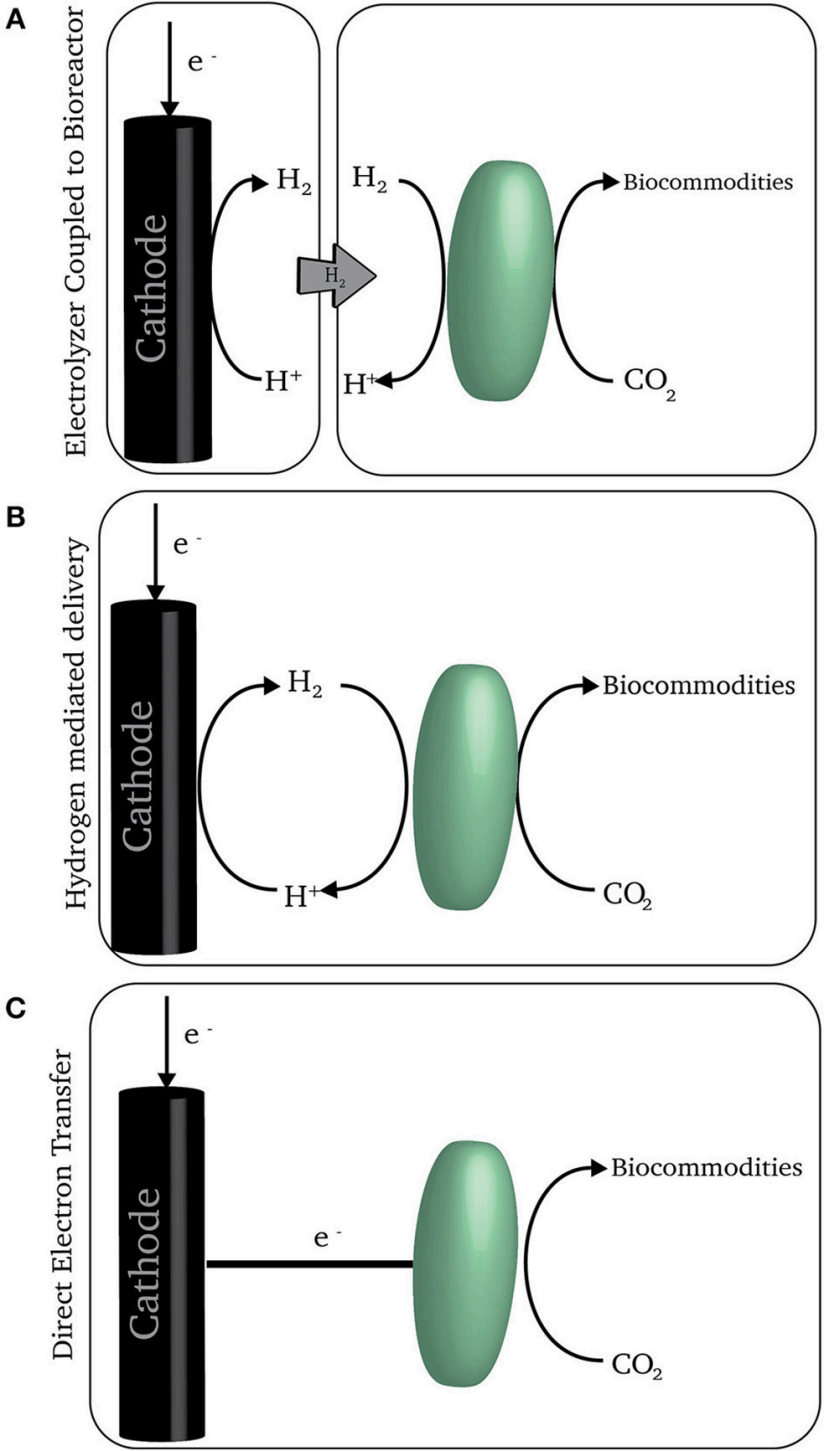
**A schematic of three electron delivery schemes. (A)** An electrolyzer coupled with a bioreactor where hydrogen is produced separately from the microorganisms consuming it. **(B)** On-demand hydrogen generation at the cathode where microorganism consume hydrogen at the point of generation. **(C)** Direct electron transfer from the cathode to microorganisms.

Therefore, methanogens and acetogens could be ideal catalysts for the conversion of excess renewable electricity to fuels and commodities, if only they could feed on electrical energy. No one has devised a strategy to incorporate microorganisms into an electrical circuit that would allow the microorganisms to directly harvest electrical energy for carbon dioxide conversion. However, there are several mechanisms by which renewable electricity can power electrochemical systems that can provide low-potential electron sources that acetogens and methanogens can use to reduce carbon dioxide to organic products that are released from the cell (Figure 2). As previously reviewed (Lovley and Nevin, 2013), there are microorganisms other than acetogens or methanogens that might be plugged into similar systems, but their efficiency of conversion of electrons to products (≤ 10%) is so low compared to the efficiency of acetogens and methanogens (≥90%) that they will not be further considered here.

Water is the largest sustainable source of electrons for feeding carbon dioxide-reducing methanogens or acetogens that has been identified to date (Lovley and Nevin, 2013). With the input of electrical energy, electrons can be liberated from water at one electrode (the anode) and donated to an electron acceptor at another electrode (the cathode). The best-known example of this electrochemical process is electrolysis yielding the overall reaction of:

(1) $$2H_{2}O\;\overset{electricalenergy}{\to }\;2H_{2}+O_{2}$$

Acetogens and methanogens evolved long ago to use H_2_ naturally present in anaerobic environments for the reduction of carbon dioxide to organic products. Thus, a simple strategy for feeding acetogens and methanogens with electrical energy is to produce H_2_ in an **electrolyzer** and then feed the H_2_ to acetogens or methanogens in a separate reactor (Varfolomeyev, 1992; Figure 2A). Alternatively, the H_2_ can be electrochemically produced, but with the cathode in the same reactor as the H_2_-consuming microorganisms (Kuroda and Watanabe, 1995; Figure 2B). Although these concepts for feeding microbes with electrochemically produced H_2_ have been around for decades, they are only now beginning to receive considerable attention.

KEY CONCEPT 1 **Direct electron transfer**

Direct electron transfer describes the extracellular exchange of electrons between a cell and an extracellular element capable of donating or receiving electrons. In this context, it describes the transfer of electrons from a cathode directly to a microbe.

KEY CONCEPT 2 **Hydrogen mediated electron delivery**

Hydrogen mediated electron delivery describes in situ hydrogen production for the on-demand consumption of microbes on the electrode surface. This abiotic production and subsequent biological oxidation of hydrogen mediates the transfer of electrons from the cathode to the microorganisms.

KEY CONCEPT 3 **Electrolyzer**

An electrolyzer is an electrochemical cell employed to split water, evolving hydrogen at a cathode and oxygen at an anode.

An alternative strategy is to directly feed electrons to microorganisms at the cathode rather than producing H_2_ (Figure 2C). In this approach the cathode is set at an electrochemical potential too positive for the production of H_2_, but sufficiently negative for microbial reduction of carbon dioxide (Nevin et al., 2010, 2011). Some acetogens may be capable of directly accepting electrons in this manner. Whether methanogens can is less certain. Methane is typically produced only at cathode potentials at which there is substantial H_2_ production (Cheng et al., 2009; Villano et al., 2010) and it is yet to be demonstrated that methanogens can directly accept electrons from a cathode (Deutzmann et al., 2015).

Each of these strategies for delivering electrical energy to microorganisms for the conversion of carbon dioxide to organic fuels or commodities has advantages and disadvantages that must be considered in designing large-scale systems. Summaries of rates of production and Coulombic efficiencies from the literature can be found in Patil et al. (2015); Blanchet et al. (2015); May et al. (2016). The purpose of this review is to consider the evolving understanding of the possibilities in reactor design for each of these approaches.

## Going old school: the electrolyzer

The concept of producing H_2_ in an electrolyzer to fuel microbial reduction of carbon dioxide (Varfolomeyev, 1992) has recently been revived (Blanchet et al., 2015). One reason for this is that H_2_ can be produced at high rates with existing electrolyzer technology, converting electrical energy into a product much faster and more efficiently than other available strategies. This decouples the hydrogen production from the microbial kinetics. Another factor is that electrochemical reactors that incorporate biology are perceived to be technically complex. Separating the electrochemistry of the electrolyzer from the biology allows each unit process to be optimized individually.

However, the electrolyzer approach comes with its own technical challenges. For example, it may be difficult to sync H_2_ production in electrolyzers and H_2_ consumption in reactors, necessitating short-term H_2_ storage. The best option is probably storage in high pressure cylinders (Götz et al., 2016).

Another challenge is the poor solubility of H_2_ (Götz et al., 2016). Multiple strategies are being investigated to make higher concentrations of H_2_ available to microorganisms in reactors, including passive delivery mechanisms like hollow fiber membranes (Ju et al., 2008; Martin and Nerenberg, 2012). In all reactor designs evaluated to date, H_2_ supply is still the factor limiting rates of methane production and further design optimization is required (Götz et al., 2016). At the present state of technological development, abiotic catalytic conversion of carbon dioxide to methane is faster than biological methane production with H_2_, requires smaller reactors and lower power requirements, and thus may be better suited for large-scale conversion of renewable electricity to methane (Götz et al., 2016).

Large-scale conversion of H_2_ to multi-carbon organic products has received less attention. Difficulties with abiotic catalysts for producing specific multicarbon organic compounds has limited this approach (Liew et al., 2013; Molitor et al., 2016). Additionally, abiotic catalysts, typically transition metals, used for the production of hydrocarbon mixtures are vulnerable to poisoning by sulfur compounds and BTEX which are often present in off-gases proposed for this type of process (Dry, 2002). Preprocessing the off-gases and post-separation of product of interest would be required, reducing the overall efficiency of production. It is more energy efficient to directly produce targeted products.

A highly controlled method for reproducibly creating specific reduced carbon compounds is to genetically modify the metabolic pathways of acetogens to eliminate the possibility of acetate production and reroute electron and carbon flow toward the production of alternative products (Ueki et al., 2014; Liew et al., 2016). With targeted genetic modification acetogens have yielded acetone, isopropanol, acetone and methylethylketone (Heijstra et al., 2013; Liew et al., 2016; Molitor et al., 2016). However, the thermodynamics of multicarbon compounds production may limited by the partial pressure of hydrogen and product titers (Agler et al., 2011).

## H_2_ from within: electrochemical production in biological reactors

Instead of producing H_2_ in a separate electrolyzer, it is possible to produce H_2_ within a biological reactor (Figure 2B). Like the electrolyzer approach, this concept has been around for decades (Kuroda and Watanabe, 1995), but was largely ignored until recently. Now, *in situ* H_2_ production is the most popular approach investigated. A concern in many of the early studies was: (1) much lower rates of H_2_ production than can be obtained with electrolyzers and (2) low recovery of electrons in desired organic products due to inefficient consumption of H_2_ (Marshall et al., 2012, 2013; Patil et al., 2015).

However, studies with a new type of cathode material have greatly accelerated the rates of *in situ* H_2_ production at the cathode while simultaneously selecting for a microbial community that can effectively consume the H_2_ as fast as it can be produced (Jourdin et al., 2016a,b). The breakthrough in cathode design was to fix carbon nanotubes on the cathode surface (Jourdin et al., 2015). For reasons that have yet to be elucidated, copper is deposited on these cathodes during microbial growth. The actual form of the copper and the mechanisms for its formation have yet to be identified and are key areas for further investigation (Jourdin et al., 2016b). Once the copper is deposited on the cathodes, it serves as a catalyst for H_2_ production even if the microorganisms are removed from the cathode (Jourdin et al., 2016b). The conversion of electrons to H_2_ with this cathode material is orders of magnitude higher than reported for other carbon-based cathode materials. Starting with an inoculum from pond sediments and an anaerobic digester, a microbial community was adapted to grow on the cathode in a biofilm that was several cell layers thick. This biofilm captures the H_2_ as fast as it can be produced. With these improvements the primary concern about the *in situ* H_2_ production approach now is whether it will be possible to design large-scale reactors. Potential strategies for scaling are discussed in a subsequent section.

## Directly feeding electrons

An alternative to producing H_2_ as an electron carrier between the cathode and microbes is to directly feed electrons to the microbes. A wide diversity of microorganisms can make direct electrical connections with electrodes (Koch and Harnisch, 2016), which at least in some cases may reflect their propensity to form syntrophic relationships to share electrons with other species (Rotaru et al., 2015). A potential advantage of direct electron feeding is that microorganisms can accept electrons for carbon dioxide reduction at more positive potentials than is required to produce H_2_. This means that less energy must be invested to make the same amount of organic product with direct electron feeding. However, even after extensive evaluation of cathode materials (Tremblay and Zhang, 2015) the rates of current conversion to organic products with direct electron feeding that have been obtained to date are much slower than the highest rates obtained with *in situ* H_2_ production. There may be opportunities to increase the rates of electron transfer from cathodes to microorganisms for direct feeding as more is learned about how microorganisms make electrical connections and this is an active area of investigation (Lovley, 2012; Malvankar and Lovley, 2014). If such advances are made direct electron feeding will still face many of the same issues with scaling up reactor size as *in situ* H_2_ production. Many design strategies and considerations for the conversion of carbon dioxide to **biocommodities** have recently been reviewed in Roy et al. (2016) and May et al. (2016).

KEY CONCEPT 4 **Biocommodities**

Biocommodities are a variety of products derived via biological pathways. In this article, many of the biocommodities referenced are potential fuel alternatives or fuel precursors. However, biocommodities more generally describe a variety of chemical and material products.

## Stripping down to scale up: the membrane-less system

**Figure 3:**
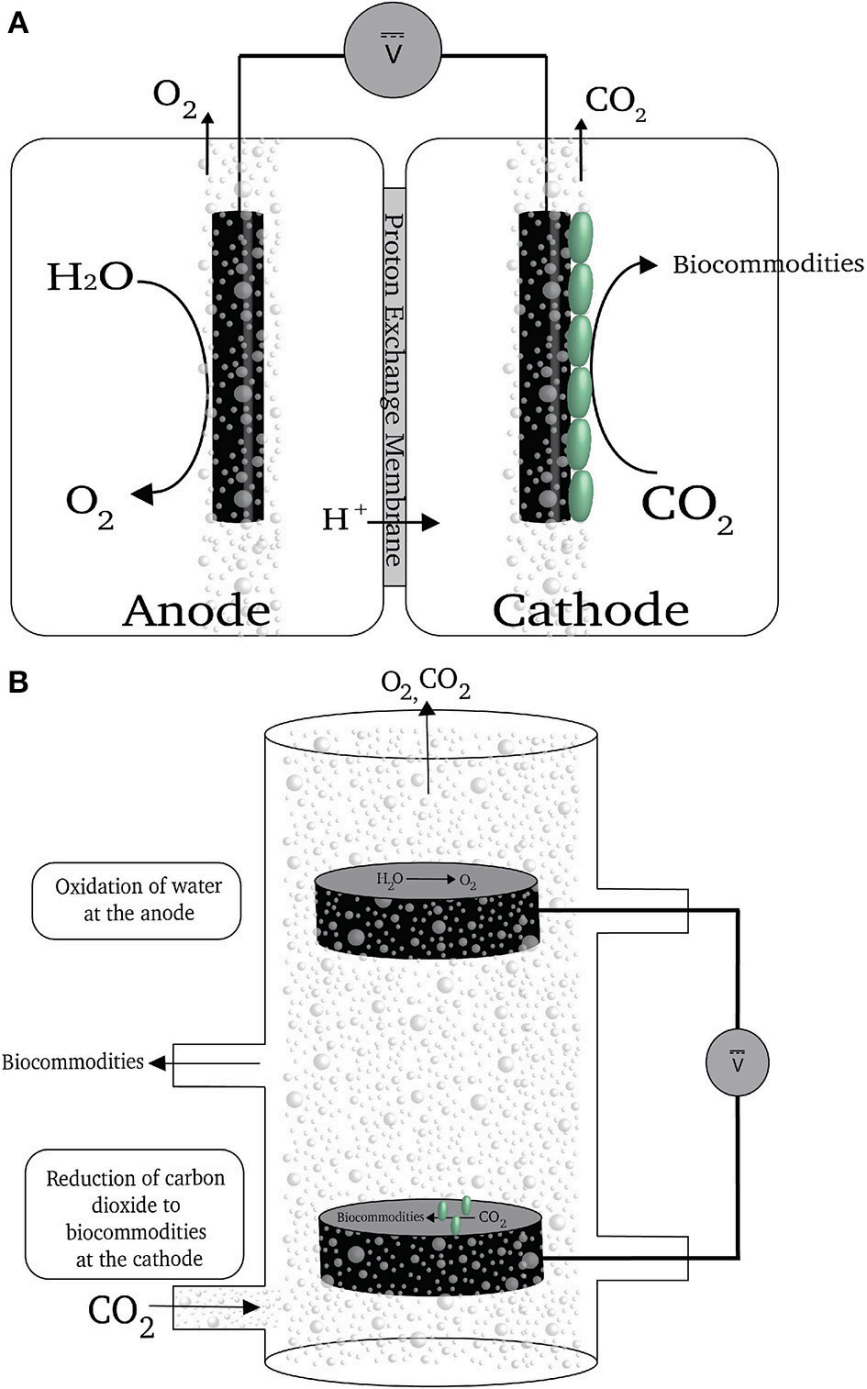
**(A)** A schematic of a reactor with a membrane partition. **(B)** A schematic of a membrane-less reactor for electrobiosynthesis of reduce carbon compounds (previously described in Giddings et al., 2015).

Most electrochemical systems that have employed microorganisms as catalysts have included membranes to separate the anode and cathode (Krieg et al., 2014). The primary role of the membranes is to impede oxygen exchange between the two chambers while permitting the passage of ions (Figure 3A). Oxygen at the cathode is detrimental regardless of whether microbes are being directly fed electrons or H_2_ is being generated because: (1) acetogens and methanogens grow best in the absence of oxygen, which can inhibit their growth and metabolism; and (2) the cathode may donate electrons to oxygen reaching the cathode surface, reducing the recovery of electrons in desired products. However, separator membranes are expensive and make reactor design more complex.

Our simple strategy to avoid the use of the membrane (Giddings et al., 2015) was to introduce the gas flow containing carbon dioxide input and the cathode near the bottom of the reactor and the oxygen-producing anode near the top of the reactor (Figure 3B). In most envisioned applications carbon dioxide will be only one component of the input gas. Therefore, even if all the carbon dioxide in the gas phase is removed as the result of microbial activity there will still be a positive upward gas flow that will strip the oxygen produced at the anode out of the reactor (Figure 3). This alleviates the need for a separator membrane to protect the microorganisms attached to the cathode. Under optimal conditions nearly 100% of the electrons consumed at the cathode were recovered in organic products in this type of reactor, demonstrating that oxygen was not negatively impacting on microbial catalysis of carbon dioxide reduction at the cathode (Giddings et al., 2015).

An additional simplification was in the electronics. Whether producing H_2_ or directly feeding microbes electrons, most reactor designs employ a type of sophisticated electronic control over the cathode that is impractical for large scale systems and is energy intensive (Rosenbaum et al., 2011). A much simpler system that is readily scalable worked just as well (Giddings et al., 2015). It is expected that coupling this simple reactor design with recently described cathode materials could make *in situ* H_2_ production a scalable technology.

## Outlook

Not surprisingly, the technology readiness levels of the three primary strategies for delivering electrons to microorganisms for carbon dioxide reduction track along with when they were first proposed. Electrolyzer technology coupled with methane production is already at the pilot scale (http://www.electrochaea.com) and though still restricted to laboratory-scale reactors, *in situ* hydrogen production is yielding faster rates of product formation than direct electron feeding.

There is little doubt one or more of these three approaches could become commercially viable as the availability of inexpensive renewal electricity continues to increase. Right now the question is whether approaches in which microorganisms are intimately associated with cathodes will provide sufficient advantages in energy efficiency, safety, operating costs, or capital costs to prevail over the electrolyzer option. Optimization of *in situ* hydrogen production will require a better understanding of the reaction(s) leading to hydrogen production at the cathode-biofilm interface and the dynamics of hydrogen consumption with cathode biofilms. There probably has been little natural selective pressure for microorganisms to rapidly consume electrons directly from possible electron donors such as other cells or reduced minerals. Therefore, enhancing rates of cathode-to-microbe electron transfer will require a much better understanding of microbe-electrode electron exchange than is currently available and, most likely, the synthetic construction of strains designed specifically for this purpose. Another question for all approaches is whether it will be feasible to generate products more valuable than methane or acetate at commercially significant rates and scales. Given the intensifying interest in these technologies, it seems likely that answers will be forthcoming. However, each of these technologies will need to compete not only with contending biological approaches, but also with rapidly emerging technologies to abiotically electrochemically convert carbon dioxide to a diversity of organic commodities (Ganesh, 2016; Götz et al., 2016). The marketplace will decide.

## Author contributions

The preparation of this review article was carried out jointly. DL and CB both contributed to review of recent literature and the written text and CB prepared the figures.

## Funding

The original collaboration noted in this review was supported by a grant from the Advanced Research Projects Agency–Energy (ARPA-E), U. S. Department of Energy, under award no. DE-AR0000087. DRL's microbial electrosynthesis research is currently supported by Office of Naval Research grant N000141310549.

### Conflict of interest statement

The authors declare that the research was conducted in the absence of any commercial or financial relationships that could be construed as a potential conflict of interest.
